# Methodologies for Assessing the Dimensional Accuracy of Computer-Guided Static Implant Surgery in Clinical Settings: A Scoping Review

**DOI:** 10.3390/dj14010043

**Published:** 2026-01-08

**Authors:** Sorana Nicoleta Rosu, Monica Silvia Tatarciuc, Anca Mihaela Vitalariu, Roxana-Ionela Vasluianu, Irina Gradinaru, Nicoleta Ioanid, Catalina Cioloca Holban, Livia Bobu, Adina Oana Armencia, Alice Murariu, Elena-Odette Luca, Ana Maria Dima

**Affiliations:** 1 Department of Oral and Maxillofacial Surgery, Grigore T. Popa University of Medicine and Pharmacy, 700115 Iasi, Romania; soranarosu@gmail.com (S.N.R.); adina.armencia@umfiasi.ro (A.O.A.); alice.murariu@umfiasi.ro (A.M.); 2 Department of Dental Prosthesis Technology, Grigore T. Popa University of Medicine and Pharmacy, 700115 Iasi, Romania; tatarciucm@yahoo.com (M.S.T.); anca.vitalariu@umfiasi.ro (A.M.V.); catalinaholban2906@gmail.com (C.C.H.); elena-odette.luca@umfiasi.ro (E.-O.L.); amadi2024@proton.me (A.M.D.); 3 Department of Prosthodontics, Grigore T. Popa University of Medicine and Pharmacy, 700115 Iasi, Romania; nicole_ioanid@yahoo.com; 4 Department of Dental Materials, Grigore T. Popa University of Medicine and Pharmacy, 700115 Iasi, Romania; irina.gradinaru@umfiasi.ro; 5 Department of Surgicals, Grigore T. Popa University of Medicine and Pharmacy, 700115 Iasi, Romania; livia.bobu@umfiasi.ro

**Keywords:** computer-aided surgery, dental implants, intraoral scanning, computer-assisted, software design

## Abstract

**Background**: Computer-guided static implant surgery (CGSIS) is widely adopted to enhance the precision of dental implant placement. However, significant heterogeneity in reported accuracy values complicates evidence-based clinical decision-making. This variance is likely attributable to a fundamental lack of standardization in the methodologies used to assess dimensional accuracy. **Objective**: This scoping review aimed to systematically map, synthesize, and analyze the clinical methodologies used to quantify the dimensional accuracy of CGSIS. **Methods**: The review was conducted in accordance with the PRISMA-ScR guidelines. A systematic search of PubMed/MEDLINE, Scopus, and Embase was performed from inception to October 2025. Clinical studies quantitatively comparing planned versus achieved implant positions in human patients were included. Data were charted on study design, guide support type, data acquisition methods, reference systems for superimposition, measurement software, and accuracy metrics. **Results**: The analysis of 21 included studies revealed extensive methodological heterogeneity. Key findings included the predominant use of two distinct reference systems: post-operative CBCT (n = 12) and intraoral scanning with scan bodies (n = 6). A variety of proprietary and third-party software packages (e.g., coDiagnostiX, Geomagic, Mimics) were employed for superimposition, utilizing different alignment algorithms. Critically, this heterogeneity in measurement approach directly manifests in widely varying reported values for core accuracy metrics. In addition, the definitions and reporting of core accuracy metrics—specifically global coronal deviation (range of reported means: 0.55–1.70 mm), global apical deviation (0.76–2.50 mm), and angular deviation (2.11–7.14°)—were inconsistent. For example, these metrics were also reported using different statistical summaries (e.g., means with standard deviations or medians with interquartile ranges). **Conclusions**: The comparability and synthesis of evidence on CGSIS accuracy are significantly limited by non-standardized measurement approaches. The reported ranges of deviation values are a direct consequence of this methodological heterogeneity, not a comparison of implant system performance. Our findings highlight an urgent need for a consensus-based minimum reporting standard for future clinical research in this field to ensure reliable and translatable evidence.

## 1. Introduction

### 1.1. Background and Rationale

The accurate three-dimensional positioning of dental implants is a crucial factor for enduring success, affecting functional, aesthetic, and biological results [1,2,3]. Deviations from the intended position can result in clinical complications, encompassing prosthetic and soft-tissue concerns, as well as grave outcomes when involving critical anatomical structures [4,5,6,7]. Computer-Guided Static Implant Surgery (CGSIS) has been widely adopted to make things more predictable. This technology sends a virtual surgical plan to the operating room using a static surgical template. This is said to improve accuracy and make the procedure easier [8]. However, the evidence base for CGSIS accuracy exhibits considerable heterogeneity, complicating clinical interpretation and comparison. Reported accuracy metrics, including global and angular deviation, exhibit significant variability across studies [9,10,11]. Clinical variables play a role, but a major reason for this spread is the wide range of methods used to measure and report accuracy [12,13,14]. This includes differences in the reference systems used to record the implant’s position, the methods used to combine planned and actual data, and the language and statistical reporting of key metrics. This methodological inconsistency represents a significant knowledge deficit, obscuring the actual efficacy of guided surgery systems. The main goals of this scoping review are to:

1. Systematically outline the methodologies utilized to assess the accuracy of CGSIS in the literature.
2. Combine the different reference systems and metrics that are used to report dimensional deviations.
3. Find out how different methodological choices can change how accurate the reported results are.
4. Identify significant gaps and sources of diversity to facilitate future research.

### 1.2. Review Question and Objectives

Given the critical need to map the methodological landscape, identify key concepts, and examine the extent, range, and nature of methodological approaches—rather than to aggregate and appraise the quantitative results of those approaches, a scoping review was selected as the most appropriate methodology. This approach is explicitly designed for clarifying complex and heterogeneous fields where varied study designs and methods are used, and where a systematic review aimed at meta-analysis would be precluded by the very inconsistency it seeks to describe. The primary research question guiding this scoping review was: What methodologies are used in the clinical literature to assess the dimensional accuracy of computer-guided static implant surgery?

To address this question, the following specific objectives were formulated:

1. To systematically map and categorize the clinical study designs employed in this domain, including randomized controlled trials, prospective cohorts, and retrospective analyses.
2. To identify, classify, and compare the reference systems (e.g., CBCT, IOS) and measurement technologies used to capture the achieved implant position and perform the planned-to-achieved superimposition.
3. To synthesize the definitions, terminology, and statistical reporting of core accuracy metrics, such as linear deviations (global, lateral, vertical) and angular deviation.
4. To identify critical gaps and inconsistencies in the current methodological approaches and, based on the synthesized evidence, propose foundational elements for a standardized reporting framework to enhance the reliability and comparability of future clinical research on CGSIS accuracy.

By achieving these objectives, this review will provide a comprehensive map of the current methodological landscape, elucidate the sources of inconsistency that pervade the literature, and propose a pathway toward a more standardized, transparent, and clinically meaningful evidence base for this highly important technology.

## 2. Methods

### 2.1. Study Design

This scoping review was conducted in accordance with the Preferred Reporting Items for Systematic reviews and Meta-Analyses extension for Scoping Reviews (PRISMA-ScR) guidelines [15]. The objective was to systematically map the literature concerning the methodologies used to assess the dimensional accuracy of computer-guided static implant surgery (CGSIS). The completed PRISMA-ScR checklist is provided in Supplementary File S1. A review protocol was not formally registered.

### 2.2. Eligibility Criteria

The inclusion and exclusion criteria were established a priori using the Population, Concept, and Context (PCC) framework, as recommended by the Joanna Briggs Institute (JBI) for scoping reviews [16]. The specific criteria are detailed in Table 1.

### 2.3. Search Strategy

A systematic literature search was performed across three major electronic bibliographic databases from their inception until October 2025: PubMed/MEDLINE, Scopus, and Embase (via Ovid). These platforms were selected for their comprehensive coverage of the biomedical and dental literature. The search strategy employed a combination of controlled vocabulary (e.g., MeSH terms) and free-text keywords related to the core concepts of “computer-guided surgery”, “dental implants”, and “accuracy”. The full search syntax for each database is available in Supplementary File S1.

### 2.4. Study Selection Process

The study selection process, illustrated in the PRISMA flow diagram (see Section 3), was conducted in two sequential phases by two independent reviewers. All identified records were collated and de-duplicated using reference management software (Zotero 6) and Microsoft Excel 365/2021. Inter-rater agreement for the full-text screening phase was calculated, showing a high level of consistency (Cohen’s kappa = 0.87).

The first step in the screening process was to compare the titles and abstracts to the PCC criteria. This step found 253 records that could be useful. The main reason for exclusion at this stage (n = 175) was that the focus was on interventions that were not part of this review, like dynamic navigation, robotic surgery, or freehand placement.

In the second phase, the complete texts of the 78 potentially pertinent studies were pursued for acquisition. Three articles could not be found. The remaining 75 studies underwent a thorough full-text review to confirm their eligibility based on the predetermined criteria. Studies were excluded if they did not primarily focus on the evaluation of CGSIS accuracy, lacked sufficient methodological details concerning the measurement procedure, or fell into an ineligible publication category (e.g., review articles). This strict process led to the final inclusion of 21 studies that directly contributed to the review’s goal by providing strong, clinically based data on how to measure dimensional accuracy. There was discussion to settle any disagreements among the reviewers until everyone agreed.

### 2.5. Data Charting Process

We used a standard data charting form made with Microsoft Excel to systematically collect data from the 21 studies that were included. During the pilot phase, definitions and categories (e.g., “CBCT-to-CBCT”, “STL-to-STL via scan bodies”) were established for complex methodological items (e.g., “reference system for superimposition”) to ensure consistent extraction. The form was validated on five randomly chosen studies and improved over time to make sure it was clear and consistent. The two reviewers acquired the data separately and then compared and resolved any differences.

The extracted data items were chosen to comprehensively address the review’s objective and included:

1. Bibliographic and General Study Information: Author, year, study design, and sample size (patients, implants, guides).
2. Surgical Protocol: Guide support type and planning software used.
3. Core Assessment Methodology: This was the focus of extraction, detailing the data acquisition method for the achieved implant position (e.g., post-operative CBCT, intraoral scan), the reference system for superimposition (e.g., planned STL vs. post-op STL), the specific software used for measurement, and the key accuracy metrics reported (e.g., linear and angular deviations).

### 2.6. Critical Appraisal of Individual Sources

Following the objective and methodological framework of a scoping review, which seeks to outline the primary concepts, evidence types, and methodologies within a discipline, a formal critical appraisal of individual sources for potential bias was not performed. This approach aligns with established practices for conducting scoping reviews. The Joanna Briggs Institute (JBI) manual for scoping reviews, for instance, says that quality assessment is not usually needed because the goal is to give a general picture of the evidence that is out there. The PRISMA extension for scoping reviews (PRISMA-ScR) also does not require a critical appraisal step. Instead, it concentrates on charting and methodically displaying the attributes of evidence. The primary objective of this review was to identify and elucidate the various methodological approaches employed in the literature, rather than to assess the strength or validity of their individual findings. Consequently, the analysis focused on meticulously defining the characteristics, scope, and distribution of the assessment methodologies employed in the included studies. This gives us a basic idea of the different ways that researchers in the field do their work. This can help with future primary research and more focused systematic reviews that include formal risk-of-bias assessments.

## 3. Results

### 3.1. Study Selection

**Figure 1:**
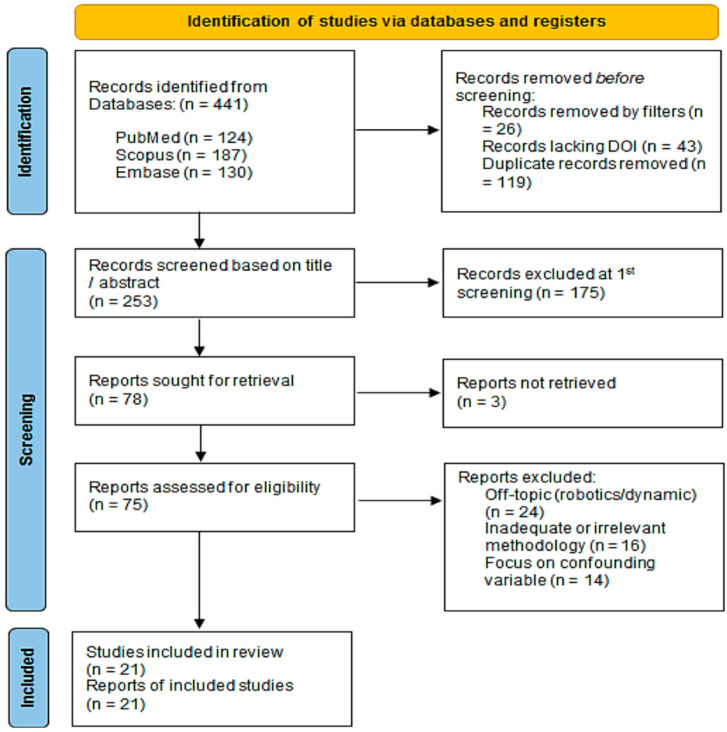
Flow diagram of the study selection process according to PRISMA ScR guidelines.

Following the PRISMA ScR guidelines, the scoping search identified 441 records from the databases. The subsequent screening and selection process is illustrated in Figure 1.

### 3.2. Characteristics of Included Studies

The 21 included studies, summarized in Table 2, were published between 2010 and 2025, reflecting the evolving nature of this field.

The sample sizes varied considerably, ranging from 5 to 59 patients, 20 to 311 implants, and 5 to 60 surgical guides. A diversity of study designs was employed: 8 were Randomized Controlled Trials (RCTs), 6 were prospective studies, and 7 were retrospective in design. The guide support types investigated were predominantly tooth-supported (n = 12 studies) and mucosa-supported (n = 13 studies), with four studies also including bone-supported guides. A wide array of planning software was utilized, with coDiagnostiX (Dental Wings) and the SimPlant/Simplant Pro suite (Materialise) being the most common.

The various study designs (RCTs, prospective/retrospective cohorts) exhibit differing degrees of methodological control, potentially influencing the robustness of accuracy estimates. Nonetheless, these designs were not subjected to formal evaluation in this scoping review.

### 3.3. Mapping of Methodological Approaches

#### 3.3.1. Reference Systems for Data Acquisition and Superimposition

The methodology for capturing the achieved implant position and the subsequent superimposition with the planned data demonstrated a fundamental dichotomy, as detailed in Table 3. This table highlights the fundamental dichotomy between CBCT-based and digital impression (IOS/STL)—based reference systems, as well as the diversity of software used for analysis.

The most common approach, used in 12 studies (57%), involved post-operative CBCT to capture the 3D position of the placed implants [18,20,23,24,25,26,29,30,31,35,36]. The reference system for these studies typically involved superimposing the pre-operative CBCT (with the virtual plan integrated) onto the post-operative CBCT. The alignment was predominantly achieved through surface-based registration of stable anatomical structures (e.g., bone, teeth) using an Iterative Closest Point (ICP) algorithm, as seen in studies using Mimics version 27.0 or coDiagnostiX software version 9.12, to perform a ”point-to-point registration” between two 3D datasets.

The second major approach, employed in 6 studies (29%), utilized digital surface data to determine the implant position [17,18,19,21,27,28]. This involved using an intraoral scanner (IOS, TRIOS, Copenhagen, Denmark) or a laboratory scanner to capture the position of a scan body connected to the implant. The reference system here was the superimposition of the STL file from the planning software (containing the virtual scan body) onto the STL file from the post-operative scan. This method relied on best-fit alignment of the scan bodies themselves or of the surrounding dentition/palate.

Three studies utilized alternative or hybrid methods. Schwindling et al. (2021) used a laboratory scan of a stone cast, while Cassetta et al. (2014) employed a sophisticated protocol to decompose total error into random and systematic components [22,32]. Sarhan et al. (2021) was unique in using fiducial markers embedded in a radiographic guide for CBCT-to-CBCT alignment [23].

#### 3.3.2. Software and Analytical Techniques

A diverse ecosystem of software was employed for the core tasks of superimposition and measurement, falling into three categories:

1. Proprietary Dental Planning Software: Used in 8 studies, these tools (e.g., coDiagnostiX, BlueSky Plan) offered integrated “treatment evaluation” modules, streamlining the workflow but potentially operating as a “black box” with undisclosed algorithms [19,20,23,25,26,29,35,36].
2. Third-party Engineering and Inspection Software: Employed in 8 studies, packages like Geomagic (Wrap, Control X, DesignX), Mimics, and MeshLab provided high flexibility and transparency in the alignment process but required advanced technical expertise [17,18,22,24,27,28,32,37].
3. Hybrid or Custom Approaches: Five studies used combinations of the above or custom algorithms, highlighting the lack of a turn-key solution [21,30,31,32,33].

#### 3.3.3. Accuracy Metrics and Statistical Reporting

In synthesizing the data from the included studies, this review uses the term “coronal deviation” to refer to linear error measured at the implant platform (also referred to in the literature as the entry point, shoulder, or cervix). “Apical deviation” refers to linear error measured at the implant apex.

The synthesis of accuracy outcomes is presented here not for direct cross-study comparison, which the methodological heterogeneity precludes, but to document the consequences of this heterogeneity on reported results and to catalog the diversity in metric definitions and statistical reporting (Table 4). The data illustrate the wide range of reported accuracy values and the heterogeneity in statistical reporting (means vs. median), which complicates cross-study comparison.

The three core metrics were nearly universally reported:

- Global Coronal Deviation: Reported in all 21 studies, with mean values ranging from 0.55 mm to 1.70 mm [19,28].
- Global Apical Deviation: Also reported in all studies, with mean values ranging from 0.76 mm to 2.50 mm [33,34].
- Angular Deviation: Reported in 20 studies, with mean values ranging from 2.11° to 11.11° (median) [23,35].

Beyond these core metrics, studies frequently decomposed deviations into directional components (e.g., mesio-distal, bucco-lingual, vertical/depth), with 12 studies reporting such data [19,20,24,25,26,29,30,32,33,34,36,37]. This practice provides more clinically actionable feedback but further adds to the complexity of cross-study comparison.

A critical source of heterogeneity was the choice of descriptive statistics. The majority of studies (n = 15) reported data as Mean ± Standard Deviation. However, a substantial number (n = 6), particularly those with smaller sample sizes or investigating non-normal distributions, reported results as Median with Interquartile Range (IQR) or Quartiles (Q1, Q3) [19,23,26,29,30,32]. This fundamental difference in data summarization presents a major challenge for any meta-analytic synthesis.

In summary, the results paint a picture of a research field utilizing robust but highly disparate methodological pathways. The choice between CBCT-based or IOS/STL-based reference systems, proprietary or third-party software, and the reporting of means versus medians creates methodological heterogeneity that prevents meaningful comparison of accuracy values across studies.

## 4. Discussion

### 4.1. Summary of Evidence

This scoping review systematically mapped the methodologies used to assess the dimensional accuracy of computer-guided static implant surgery (CGSIS) across 21 clinical studies. The principal finding reveals profound methodological heterogeneity, which permeates every stage of the assessment workflow. This lack of standardization constitutes a fundamental issue that confounds the entire evidence base. Our analysis highlights significant variations in study designs, a core dichotomy in the reference systems for capturing achieved implant position (CBCT-based versus digital impression-based), a diverse array of software for superimposition and measurement, and inconsistent definitions and statistical reporting of core accuracy metrics. Collectively, these methodological disparities offer a clear, evidence-based explanation for the widely dispersed accuracy values present in the existing literature.

### 4.2. Interpretation of Key Findings

#### 4.2.1. The Dichotomy of Reference Systems. Mapping Distinct Methodological Paradigms and Their Clinical Constructs

This review mapped two dominant, distinct methodological paradigms for assessing accuracy, each designed to answer a different clinical question and measuring a different construct.

This analysis reveals two dominant, yet fundamentally different, pathways for assessing clinical accuracy: post-operative CBCT and intraoral scanning (IOS) with scan bodies. It is important to interpret the substantial and systematic variation in reported numerical values primarily as a direct illustration of these methodological differences, rather than as a basis for direct clinical comparison or inference. Each pathway answers a distinct clinical question and carries inherent limitations that directly and predictably shape its results.

The CBCT-based approach assesses the bone-level implant position. While it provides a direct visualization of the implant within the bone, its accuracy is challenged by a lack of standardization. Scatter artifacts from the implant and adjacent structures can obscure the true implant axis, a methodological limitation that likely contributes to the elevated angular deviation values reported in studies such as Wang et al. (2024) and Sarhan et al. (2021) [18,23]. Consequently, the dispersion in CBCT-derived data must be interpreted in light of these inherent measurement constraints.

In contrast, the IOS-based approach benefits from the high precision of optical scanning, which is not susceptible to radiographic scatter [17,19,21,27,28]. This method captures the prosthetic platform with high fidelity, as demonstrated by studies like Luongo et al. (2024) and Monaco et al. (2020), which reported lower mean global coronal deviations (0.73 mm and ~0.92 mm, respectively) [17,25]. However, this method infers the apical position based on the planned implant axis, failing to account for any bending or deflection of the implant during insertion. It measures prosthetic-driven accuracy but does not validate the bone-level position, which is important for assessing proximity to vital structures. The choice of system, therefore, answers different clinical questions, and their results are not directly comparable (Table 5).

Recent investigations reinforce that this dichotomy is not merely binary but contains further methodological layers. For example, Limones et al. (2024) demonstrated that within the IOS pathway, the alignment method (e.g., using a single versus multiple scan bodies) can significantly alter the reported deviation values [38]. This underscores that the observed dispersion in data is often a direct artifact of the chosen assessment protocol. Furthermore, a novel non-radiologic method (digital registration method), which uses laboratory scans of physical casts with implant analogs, has been validated as comparable to CBCT-based assessment, offering a third, radiation-free pathway for certain cases [39,40,41,42].

In vitro study by Pellegrino et al. (2022) directly compared three assessment methods (CBCT, laboratory scanner, IOS) and found that while the operator’s experience significantly influenced accuracy, the choice of evaluation method also yielded statistically different results, underscoring that methodology itself is a variable that can confound performance comparisons [43].

Therefore, these pathways are not interchangeable, and their results represent distinct constructs, bone-level versus restorative-level accuracy, that should not be directly compared.

#### 4.2.2. The Problem of Inconsistent Terminology and Statistical Reporting

Recent investigations suggest that this dichotomy is not solely binary but encompasses additional methodological aspects. For instance, Limones et al. (2024) illustrated that within the IOS pathway, the alignment method (e.g., employing a single versus multiple scan bodies) can significantly modify the reported deviation values [38]. This demonstrates that the perceived variability in data frequently results directly from the selected assessment protocol. Additionally, a new non-radiologic technique (digital registration method) that employs laboratory scans of physical casts with implant analogs has been confirmed as equivalent to CBCT-based evaluation, providing a third, radiation-free option for specific instances [44].

Furthermore, there is a widespread conflation of the ISO-defined terms trueness (deviation from a true value) and precision (reproducibility). Most clinical studies measure trueness—the deviation from the plan. However, without repeated measurements to assess precision, the reliability of a system’s performance remains unknown. Adhering to this standardized terminology would bring much-needed clarity.

Recent literature confirms the persistent use of heterogeneous metrics. For instance, Mittal et al. (2025) reported deviations with means and standard deviations, while Song et al. (2025) also used means and SDs but focused on “trueness”, correctly adopting the ISO term [45,46,47]. This represents a small step towards standardization in terminology, though statistical reporting remains inconsistent.

#### 4.2.3. The Impact of Clinical Reality on Measured Accuracy

The extracted data consistently illustrate that clinical complexity is a primary determinant of reported accuracy, a factor that must be contextualized within the aforementioned methodological frameworks. Controlled scenarios in partially edentulous cases yield lower deviations [17,19,27], while challenging clinical conditions—such as mucosa-supported guides in edentulous arches—consistently result in higher reported inaccuracies [18,23,30].

This pattern quantitatively validates that clinical variables (e.g., guide fit, tissue resilience) are significant, measurable sources of error [32]. Recent studies further dissect these variables, showing that factors like guide manufacturing method, sterilization, and surgical cantilever length have a quantifiable and significant impact on the final deviation values [48,49]. Therefore, the dispersion in the evidence base should be understood as a combined function of (a) the chosen measurement pathway’s inherent limitations, (b) inconsistent data reporting, and (c) the genuine spectrum of clinical difficulty. Isolating the clinical signal from the methodological noise remains a central challenge in interpreting this body of literature.

### 4.3. Implications for Practice and Research

#### 4.3.1. For Researchers: Proposed Framework

To mitigate the identified heterogeneity and foster a more cumulative science, based on the evidence mapped in this review, we propose a conceptual framework for a Minimum Reporting Standard for future clinical studies on CGSIS accuracy. We emphasize that this is a proposal derived from the synthesis of current methodological inconsistencies, intended to spark discussion and consensus-building, and not a formally endorsed guideline. This framework suggests that future studies aim to report the following elements:

1. Explicit Methodology Description: A detailed account of the reference system, including the specific hardware (CBCT machine, IOS model) and software (name and version) used for superimposition and measurement, including the alignment algorithm (e.g., ICP, best-fit).
2. Should include Core Metrics: Reporting of the following three metrics for every implant, as a minimum:
  - Global 3D deviation at the implant platform (coronal);
  - Global 3D deviation at the implant apex;
  - Three-dimensional angular deviation.
3. Standardized Statistical Reporting: Providing both mean ± standard deviation and median with interquartile range (IQR) for all core metrics to accommodate both parametric and non-parametric understanding of the data.
4. Adherence to ISO Terminology: Clearly stating that the study is assessing trueness and, where possible, designing studies to also evaluate precision.
5. Reporting of Clinical Confounders: Should include reporting of key clinical variables known to affect accuracy, including but not limited to: guide support type, edentulism status (fully vs. partially), surgical protocol (fully vs. partially guided), implant region (anterior/posterior), and use of anchor pins.

#### 4.3.2. For Clinicians: A Critical Lens for Interpreting the Evidence

Clinicians must interpret published accuracy data with a critical understanding of the methodology employed. A reported accuracy value is not an absolute truth but a measurement contingent on the assessment protocol. When evaluating a system or study, clinicians should ask: Was the achieved position captured by CBCT or IOS? What was the guide support type? The answers to these questions provide essential context. For instance, a low deviation value from an IOS-based study in a tooth-supported case may not be transferable to the clinical reality of a fully edentulous, mucosa-supported rehabilitation. Understanding these methodological nuances is essential to forming realistic expectations and making evidence-based choices.

The emergence of dynamic navigation and robotic-assisted surgery (r-CAIS) provides an important new context. Multiple recent studies consistently show that r-CAIS achieves significantly higher accuracy than s-CAIS [50,51,52,53]. For instance, a clinical study on fully edentulous jaws reported high accuracy for dynamic navigation, a technology whose assessment methodology faces similar standardization challenges [54]. Chen et al. (2025) reported mean angular deviations of 1.51 for robotics versus 3.44 for static guides [46]. When reading the literature, clinicians must now differentiate between these fundamentally different guided technologies, as the performance benchmarks are shifting.

### 4.4. Limitations of the Review

While this review provides a comprehensive map of the methodological landscape, certain limitations must be acknowledged. The search was restricted to three major databases, and although no language restrictions were applied, it is possible that relevant studies in other databases or in the gray literature were missed. The field of digital dentistry is evolving at a rapid pace; new planning software, scanning technologies, and analysis tools are continuously emerging. Furthermore, the eligibility criteria required accuracy to be the primary outcome with detailed methodological description. This necessarily excluded studies where accuracy was a secondary outcome or reported with less detail, which may limit the depiction of reporting practices in the broader clinical literature. This review represents a snapshot in time, and the methodological trends we identified may shift with future technological advancements. Finally, as a scoping review, our objective was to map the evidence rather than appraise the quality of individual studies, which means our conclusions speak to the collective practices of the field rather than the validity of any single finding.

## 5. Conclusions

This scoping review demonstrates that clinical literature on the dimensional accuracy of Computer-Guided Static implant Surgery (CGSIS) is marked by extensive methodological heterogeneity. The direct comparison of accuracy values across studies is hindered by inconsistent reference systems, diverse measurement software, and variable reporting of metrics and statistics. Consequently, reported outcomes are highly dependent on the specific assessment protocol used. To facilitate the generation of comparable, reliable, and clinically meaningful evidence, this review highlights the necessity for standardized methodology and reporting in CGSIS accuracy research and suggests key elements to inform future consensus-building efforts. The reported performance of CGSIS is inextricably linked to the assessment protocol used, rather than reflecting the technology’s intrinsic capability alone.

## Figures and Tables

**Table 1:** Study Inclusion and Exclusion Criteria.

PCC Element	Inclusion Criteria	Exclusion Criteria
Population	Human patients receiving dental implants placed exclusively via computer-guided static implant surgery (CGSIS).	In vitro studies, cadaveric studies, animal studies, and studies involving dynamic navigation or robotic surgery
Concept	Primary clinical studies quantitatively comparing the planned versus the achieved 3D implant position. Required detailed description of the assessment methodology, including data acquisition, reference system for superimposition, and measurement software.	Studies where accuracy assessment was not the primary outcome, or where the methodology for measuring deviation was not described.
Context	Primary research published in peer-reviewed journals.	Case reports (<5 patients), technical notes, narrative reviews, systematic reviews, meta-analyses, and conference abstracts.

**Table 2:** Characteristics of Included Studies.

Authors (Year)	Study Design	Sample Size (Patients/Implants/Guides)	Guide Support Type	Planning Software
Luongo et al. (2024) [17]	Multicenter Retrospective Clinical Study	21/61/21 (one guide per patient)	Tooth-supported (for partial/single edentulism) and Mucosa-supported (for total edentulism)	RealGUIDE 5.0™, 3Diemme, Cantù, Italy)
Wang et al. (2024) [18]	Retrospective Study	24/120/28	Mucosa-supported; Mucosa + Tooth-supported	Specialized software from Hangzhou 6D DENTAL, Hangzhou 6D DENTAL Technology Co., Ltd., Hangzhou, China)
Sun et al. (2022) [19]	Randomized Controlled Trial (RCT)	28/30/30	Tooth-supported (Test: 2 teeth; Control: 3 teeth)	coDiagnostiX 9, Dental Wings GmbH, Chemnitz, Germany)
Ngamprasertkit et al. (2022) [20]	Randomized Controlled Trial (RCT)	30/30/30 (15 Surgical Drill Guide (SDG), 15 Surgical Drill and Implant Guide (SDIG)	Tooth-supported	Planmeca Romexis™, Planmeca Oy, Helsinki, Finland
Orban et al. (2022) [21]	Prospective, Randomized Controlled Trial (RCT)	40/40/40	Tooth-supported	SMART Guide, dicomLAB Dental, Houten, Netherlands
Schwindling et al. (2021) [22]	Prospective Clinical Feasibility Study (Registered: DRKS00014239)	27/41/27 (34 patients enrolled, 7 dropouts; 41 implants in 27 patients; one guide per patient)	Tooth-supported	CoDiagnostiX 9.12, Dental Wings Inc., Montreal, Canada)
Sarhan et al. (2021) [23]	Split-Mouth Clinical Study	12/48/12	Mucosa-supported (tissue-supported)	Blue Sky Bio, Blue Sky Bio, LLC, Libertyville, IL, USA
Chai et al. (2020) [24]	Prospective Cohort	9/44/12	Mucosa-supported	Organical^®^ Dental Implant (ODI 1.1.0.5), Organical Ltd., Tel Aviv, Israel
Monaco et al. (2020) [25]	Retrospective Study	56/120/56	Tooth-supported (Fully Guided with Adequate support (FGA), Fully Guided with Minimum support (FGM), Partially Guided (PG) groups); Mucosa-supported (MS group)	SMOP software, Swissmeda AG, Zurich, Switzerland)
Kiatkroekkrai et al. (2020) [26]	Randomized Controlled Trial (RCT)	47/60/60	Tooth-supported (cross-arch stabilization)	coDiagnostiX 9.7 (Dental Wings Inc.), Canada
Smitkarn et al. (2019) [27]	Randomized Controlled Trial (RCT)	52/60/30 (1 guide per CAIS patient)	Tooth-supported	coDiagnostiX^®^ v9.7 (by association with Dental Wings Inc., Montreal, Canada
Younes et al. (2018) [28]	Randomized Controlled Trial (RCT)	32/71/21 (10 FG guides + 11 PG guides)	Tooth-supported	Simplant^®^ 17.0, Materialise Dental, Leuven, Belgium
Schnutenhaus et al. (2016) [29]	Retrospective Study	24/24/24	Tooth-supported (with a distal gingival contact for DES cases)	SMOP v2.6, Swissmeda AG, Zurich, Switzerland
Vercruyssen et al. (2014) [30]	Randomized Controlled Trial (RCT)	59 patients/311 implants/48 guides (12 jaws per group × 4 guided groups)	Bone-supported; Mucosa-supported	Simplant^®^ 17.0, Materialise Dental, Leuven, Belgium
Verhamme et al. (2013) [31]	Prospective Clinical Study	5/20/5	Mucosa-supported	Procera Clinical Design, Nobel Biocare Services AG, Zurich, Switzerland
Cassetta et al. (2014) [32]	Observational, Retrospective Study	24/172/18	Mucosa-supported, fixed with osteosynthesis screws	SimPlant, Materialise Dental, Leuven, Belgium
Arısan et al. (2010) [33]	Clinical Comparative Study	54/294/60	Bone-supported; Tooth-supported; Mucosa-supported	SimPlant (Materialise) & NobelGuide (Nobel Biocare), Leuven, Belgium & Zurich, Switzerland
Kraft et al. (2020) [34]	Randomized Controlled Trial (RCT)	24/24/24	Tooth-supported	coDiagnostiX, DentalWings GmbH, Chemnitz, Germany
Alqutaibi et al. (2025) [35]	Split-Mouth Randomized Controlled Trial	16/96/16	Mucosa-supported	BlueSkyPlan 4, Blue Sky Bio, LLC, Libertyville, IL, USA
D’addazio et al. (2022) [36]	Prospective Clinical Study (In Vivo)	10/48/10 (5 patients per group; 24 implants per group)	Mucosa-supported	Proprietary to Nobel Biocare & GEASS protocols, Nobel Biocare, Zurich, Switzerland & GEASS, Torre San Giorgio, Italy
Cassetta et al. (2013) [37]	Retrospective Clinical Study	12/129/18	Mucosa-supported (103), Bone-supported (18), Teeth-supported (8)	SimPlant^®^, Materialise Dental, Leuven, Belgium

**Table 3:** Methodologies for Accuracy Assessment.

Authors (Year)	Data Acquisition (Achieved Position)	Reference System (Datasets Superimposed)	Measurement/Superimposition Software
Luongo et al. (2024) [17]	Intraoral Scan (IOS) with scan body (CS 3600^®^ or i500^®^ scanner)	Planned STL (from planning software with scanbody) vs. Post-op IOS STL (with scanbody in place)	GeoMagic Wrap 12^®^ for “Best-fit Alignment” based on anatomical areas (teeth/palate), 3D system, Rock Hill, SC, USA.
Wang et al. (2024) [18]	Post-operative CBCT	Pre-op CBCT (DICOM) + Planned STL vs. Post-op CBCT (DICOM)	Mimics 21.0 & 3-Matic 13.0, Materialise, Leuven, Belgium
Sun et al. (2022) [19]	Intraoral Scan (IOS) with scan body (TRIOS Standard, 3Shape)	Planned STL vs. Post-op IOS STL	coDiagnostiX software (using point-to-point registration),Dental Wings Inc., Montreal, QC, Canada
Ngamprasertkit et al. (2022) [20]	Post-operative CBCT	Planned (from pre-op CBCT) vs. Post-op CBCT	Planmeca Romexis™ (for superimposition) Planmeca, Helsinki, Finland, Image J (for measurement), NIH, Bethesda, MD, USA
Orban et al. (2022) [21]	Intraoral Scan (IOS) with scan body (Planmeca PlanScan)	Pre-op CBCT DICOM (with planning) vs. Post-op IOS STL	Amira 5.4.0 with dicomLAB Dental, Thermo Fisher Scientific, Hillsboro, OR, USA
Schwindling et al. (2021) [22]	Laboratory Scan of Stone Cast (from high-precision polyether impression with scan bodies)	Planned STL (from dMRI) vs. Post-op STL (from cast scan)	Superimposition & Analysis: Geomagic DesignX, 3D Systems, Rock hill, SC, USA. Scanning: D2000 laboratory scanner, 3Shape, Copenhagen, Denmark. Mesh Deviation (RMS): 337 ± 98 µm.
Sarhan et al. (2021) [23]	Post-operative CBCT	Pre-op CBCT (with radiographic guide) vs. Post-op CBCT (with surgical guide in place), aligned using fiducial markers.	Blue Sky Bio, Blue Sky Bio LLC, Libertyville, IL, USA
Chai et al. (2020) [24]	Post-operative CBCT	Pre-op CBCT (with virtual plan) vs. Post-op CBCT	Mimics 19.0 for 3D model generation from DICOM, Materialise, Leuven, Belgium Organical^®^ Dental Implant for superimposition and measurement, Organical CAD/CAM GmbH, Berlin, Germany
Monaco et al. (2020) [25]	Intraoral Scan (IOS) with scan body	Planned STL vs. Post-op IOS STL	MeshLab software ISTI-CNR, Visual Computing Lab, Pisa, Italy
Kiatkroekkrai et al. (2020) [26]	Post-operative CBCT (3D Accuitomo 170 machine)	Planned (from pre-op CBCT & surface scan) vs. Post-op CBCT	coDiagnostiX 9.7 (Treatment evaluation tool function), Canada
Smitkarn et al. (2019) [27]	Post-operative CBCT	Planned (from pre-op CBCT) vs. Post-op CBCT	coDiagnostiX^®^ v9.7 Automated surface best fit with iterative closest point algorithm, Dental Wings Inc., Montreal, QC, Canada
Younes et al. (2018) [28]	Post-operative CBCT	Pre-op CBCT (with virtual plan) vs. Post-op CBCT	1. Simplant Pro for initial data processing, bone model creation, and initial registration, Materialise Dental, Leuven, Belgium 2. Magics for extracting planned implant coordinates, Materialise, Leuven, Belgium 3. Mimics for creating 3D models of placed implants and final coordinate extraction, Materialise, Leuven, Belgium
Schnutenhaus et al. (2016) [29]	Laboratory Scan of Master Cast (from a polyether open-tray impression with implant analogs)	Planned STL (from SMOP) vs. Achieved STL (from master cast scan)	Superimposition: Geomagic Studio 9 Deviation Analysis: Geomagic Qualify 9 & Surfacer 10.6, 3D Systems, Rock Hill, SC, USA
Vercruyssen et al. (2014) [30]	Post-operative Cone Beam CT (CBCT)	Pre-operative MSCT (Planning Data) vs. Post-operative CBCT	Mimics^®^ (Materialise Dental) using an iterative closest point (ICP) algorithm for surface-based registration, Materialise, Leuven, Belgium
Verhamme et al. (2013) [31]	Post-operative CBCT	Pre-op CBCT (with planned implants) vs. Post-op CBCT	NobelGuide Validation Software (Medicim NV) + IPOP Method, Medicim NV, Mechelen, Belgium
Cassetta et al. (2014) [32]	Post-operative Computed Tomography (CT)	For Total Error: Pre-op CT (jaw) vs. Post-op CT (jaw). For Random Error: Pre-op CT (planned implants) vs. Post-op CT (placed implants). Systematic Error: Calculated as (Total Error–Random Error).	Mimics with an Iterative Closest Point (ICP) algorithm, Materialise, Leuven, Belgium
Arısan et al. (2010) [33]	Post-operative CBCT	Pre-op Planning vs. Post-op CBCT	Mimics (for image fusion); Planning software (for measurement),Materialise, Leuven, Belgium
Kraft et al. (2020) [34]	Post-operative CT Scan	Planned (from pre-op CT & IOS) vs. Post-op CT	coDiagnostiX, Dental Wings Inc., Montreal, QC, Canada
Alqutaibi et al. (2025) [35]	Post-operative CBCT (I-CAT)	Planned (from pre-op CBCT) vs. Post-op CBCT	BlueSky Software, BlueSky Bio LLC, Libertyville, IL, USA
D’addazio et al. (2022) [36]	Post-operative CBCT	Pre-operative Virtual Planning STL vs. Post-operative CBCT (converted to STL)	Geomagic (Best-fit algorithm for superimposition), 3D Systems, Rock Hill, SC, USA
Cassetta et al. (2013) [37]	Post-operative CT	Pre-op CT vs. Post-op CT	SimPlant^®^ using an Iterative Closest Point algorithm, Materialise Dental, Leuven, Belgium

**Table 4:** Reported Accuracy Outcomes.

Authors (Year)	Key Accuracy Metrics Reported (Mean ± SD, Unless Otherwise Noted)
Luongo et al. (2024) [17]	Angular: 2.94° ± 1.84°
	Coronal 3D (Linear Platform): 0.73 ± 0.30 mm
	Apical 3D (Linear Apex): 1.06 ± 0.53 mm
	Vertical (Platform): 0.29 ± 0.44 mm
	Vertical (Apex): 0.01 ± 0.78 mm
	Barycenter Deviation: 0.67 ± 0.47 mm
Wang et al. (2024) [18]	Global Coronal: 1.53 ± 0.65 mm; Global Apical: 1.91 ± 0.68 mm; Angular: 7.14 ± 3.41°; Coronal Lateral: 0.98 ± 0.53 mm; Coronal Vertical: 1.01 ± 0.69 mm; Apical Lateral: 1.47 ± 0.68 mm; Apical Vertical: 1.02 ± 0.69 mm
Sun et al. (2022) [19]	Angular: 4.23 ± 2.38° (Test) vs. 4.13 ± 2.42° (Control); Coronal 3D: 0.70 ± 0.44 mm vs. 0.55 ± 0.27 mm; Apical 3D: 1.25 ± 0.61 mm vs. 1.11 ± 0.54 mm; Buccolingual (Apex): 0.94 ± 0.53 mm vs. 0.85 ± 0.42 mm. Median (Q1, Q3) for non-parametric data: Mesiodistal (Shoulder): 0.14 (0.07, 0.28) mm vs. 0.12 (0.02, 0.26) mm; Buccolingual (Shoulder): 0.26 (0.18, 0.35) mm vs. 0.34 (0.14, 0.59) mm; Apicocoronal (Shoulder): 0.31 (0.25, 0.77) mm vs. 0.19 (0.10, 0.40) mm; Mesiodistal (Apex): 0.35 (0.13, 0.74) mm vs. 0.41 (0.12, 0.58) mm; Apicocoronal (Apex): 0.31 (0.24, 0.75) mm vs. 0.16 (0.12, 0.38) mm.
Ngamprasertkit et al. (2022) [20]	Global Deviation (Shoulder): 0.74 ± 0.36 mm Surgical Drill Guide (SDG) vs. 0.48 ± 0.22 mm Surgical Drill and Implant Guide (SDIG)
	Global Deviation (Apex): 1.29 ± 0.61 mm (SDG) vs. 0.71 ± 0.31 mm (SDIG)
	Horizontal Deviation (Shoulder): 1.17 ± 0.68 mm (SDG) vs. 0.64 ± 0.37 mm (SDIG)
	Vertical Deviation (Shoulder): 0.37 ± 0.27 mm (SDG) vs. 0.20 ± 0.13 mm (SDIG)
	Horizontal Deviation (Apex): 1.17 ± 0.68 mm (SDG) vs. 0.64 ± 0.37 mm (SDIG)
	Vertical Deviation (Apex): 0.37 ± 0.27 mm (SDG) vs. 0.20 ± 0.13 mm (SDIG)
	Angular Deviation: 4.03 ± 1.95° (SDG) vs. 2.45 ± 1.32° (SDIG)
Orban et al. (2022) [21]	Angular Deviation: 4.82 ± 2.07° (Machine) vs. 4.11 ± 1.63° (Manual)
	Global Coronal Deviation: 1.20 ± 0.46 mm vs. 1.13 ± 0.38 mm
	Global Apical Deviation: 1.45 ± 0.79 mm vs. 1.18 ± 0.28 mm
	Horizontal Coronal Deviation: 1.06 ± 0.52 mm vs. 0.92 ± 0.40 mm
	Horizontal Apical Deviation: 1.28 ± 0.83 mm vs. 0.99 ± 0.28 mm
	Vertical Deviation (Apical): 0.55 ± 0.28 mm vs. 0.62 ± 0.21 mm
Schwindling et al. (2021) [22]	For implants placed as initially planned in dMRI (n = 28):
	Angular Deviation: 7.1 ± 4.8°
	Coronal 3D Deviation: 1.7 ± 0.9 mm
	Apical 3D Deviation: 2.3 ± 1.1 mm
Sarhan et al. (2021) [23]	Reported as Median (IQR): Horizontal Deviation at Hexagon: 1.65 mm (0.91–4.54) [Fully Guided Cylindrical] Horizontal Deviation at Apex: 1.91 mm (0.77–6.68) [Fully Guided Cylindrical] Angular Deviation: 11.11° (4.22–14.76) [Fully Guided Cylindrical] Apical Depth Deviation: 1.01 mm (0.28–2.54) [Fully Guided Cylindrical] No statistically significant differences were found between Fully Guided vs. Partially Guided protocols, nor between Cylindrical vs. C-shaped guide holes for any of these metrics.
Chai et al. (2020) [24]	Global Coronal Deviation: 1.53 ± 0.48 mm
	Global Apical Deviation: 1.58 ± 0.49 mm
	Angular Deviation: 3.96 ± 3.05°
	Horizontal Deviation (Coronal): 1.33 ± 0.50 mm
	Horizontal Deviation (Apical): 1.37 ± 0.52 mm
	Depth Deviation (Coronal): 0.50 ± 0.38 mm
	Depth Deviation (Apical): 0.51 ± 0.40 mm
	Mean Inter-implant Distance Difference: 0.48 ± 0.51 mm (coronal), 0.50 ± 0.43 mm (apical)
Monaco et al. (2020) [25]	Global 3D Deviation (Head): 0.92 ± 0.52 mm Fully Guided with Adequate support (FGA), 0.91 ± 0.44 mm Fully Guided with Minimum support (FGM), 0.95 ± 0.47 mm Partially Guided (PG), 1.15 ± 0.45 mm (MS)
	Global 3D Deviation (Apex): 1.14 ± 0.54 mm (FGA), 1.11 ± 0.54 mm (FGM), 1.17 ± 0.49 mm (PG), 1.42 ± 0.45 mm (MS)
	Angular Deviation: 2.45 ± 1.24° (FGA), 2.73 ± 1.96° (FGM), 3.71 ± 1.67° (PG), 4.19 ± 2.62° (MS)
	2D Lateral Deviation (Head): 0.57 ± 0.38 mm (FGA), 0.59 ± 0.39 mm (FGM), 0.62 ± 0.42 mm (PG), 0.70 ± 0.46 mm (MS)
	2D Lateral Deviation (Apex): 0.76 ± 0.45 mm (FGA), 0.74 ± 0.44 mm (FGM), 0.78 ± 0.47 mm (PG), 0.94 ± 0.50 mm (MS)
Kiatkroekkrai et al. (2020) [26]	Angular: 2.41° ± 1.47° (IO) vs. 3.23° ± 2.09° (Model); Coronal 3D: 0.87 ± 0.49 mm vs. 1.01 ± 0.56 mm; Apical 3D: 1.10 ± 0.53 mm vs. 1.38 ± 0.68 mm; Mesiodistal (Coronal): 0.35 ± 0.30 mm vs. 0.38 ± 0.37 mm; Buccolingual (Coronal): 0.32 ± 0.35 mm vs. 0.41 ± 0.36 mm; Vertical (Coronal): 0.58 ± 0.47 mm vs. 0.69 ± 0.54 mm; Mesiodistal (Apical): 0.52 ± 0.45 mm vs. 0.63 ± 0.64 mm; Buccolingual (Apical): 0.54 ± 0.41 mm vs. 0.74 ± 0.52 mm; Vertical (Apical): 0.59 ± 0.48 mm vs. 0.69 ± 0.54 mm.
Smitkarn et al. (2019) [27]	Median (IQR) for Static CAIS vs. Freehand: Angular Deviation: 2.8 (2.6)° vs. 7.0 (7.0)° Coronal 3D Deviation: 0.9 (0.8) mm vs. 1.3 (0.7) mm Apical 3D Deviation: 1.2 (0.9) mm vs. 2.2 (1.2) mm Coronal Mesiodistal: 0.2 (0.3) mm vs. 0.6 (0.5) mm Coronal Buccolingual: 0.3 (0.4) mm vs. 0.4 (0.5) mm Coronal Apicocoronal: 0.6 (0.6) mm vs. 0.9 (0.7) mm Apical Mesiodistal: 0.5 (0.6) mm vs. 1.1 (1.3) mm Apical Buccolingual: 0.6 (0.8) mm vs. 0.7 (1.6) mm Apical Apicocoronal: 0.6 (0.6) mm vs. 0.7 (0.8) mm
Younes et al. (2018) [28]	Apical Global Deviation (AGD): 0.97 mm (FG) vs. 1.43 mm (PG) vs. 2.11 mm (FH)
	Coronal Global Deviation (CGD): 0.73 mm (FG) vs. 1.12 mm (PG) vs. 1.45 mm (FH)
	Angular Deviation (AD): 2.30° (FG) vs. 5.95° (PG) vs. 6.99° (FH)
	Coronal Lateral Deviation (CLD): 0.55 mm (FG) vs. 0.79 mm (PG) vs. 1.27 mm (FH)
	Apical Lateral Deviation (ALD): 0.81 mm (FG) vs. 1.14 mm (PG) vs. 1.97 mm (FH)
	Coronal Vertical Deviation (CVD): 0.43 mm (FG) vs. 0.68 mm (PG) vs. 0.53 mm (FH)
	Apical Vertical Deviation (AVD): 0.43 mm (FG) vs. 0.68 mm (PG) vs. 0.50 mm (FH)
Schnutenhaus et al. (2016) [29]	DES Group (n = 12): Angular Deviation (α): 5.0° ± 3.1° Coronal 3D (d1): 1.0 ± 0.5 mm Apical 3D (d2): 1.6 ± 0.7 mm Vertical (Height, h): 0.5 ± 0.7 mm STG Group (n = 12): Angular Deviation (α): 4.0° ± 1.5° Coronal 3D (d1): 0.9 ± 0.5 mm Apical 3D (d2): 1.5 ± 0.7 mm Vertical (Height, h): 0.5 ± 0.6 mm
Vercruyssen et al. (2014) [30]	Guided Surgery (Pooled, n = 209 implants): Coronal 3D: 1.4 mm (Range: 0.3–3.7 mm) Apical 3D: 1.6 mm (Range: 0.2–3.7 mm) Angular: 3.0° (Range: 0.2–16.0°) By Group (Mean ± SD): Mat Mu: Coronal: 1.23 ± 0.60 mm; Apical: 1.57 ± 0.71 mm; Angular: 2.86 ± 1.60° Mat Bo: Coronal: 1.60 ± 0.92 mm; Apical: 1.65 ± 0.82 mm; Angular: 3.79 ± 2.36° Fac Mu: Coronal: 1.38 ± 0.64 mm; Apical: 1.60 ± 0.70 mm; Angular: 2.71 ± 1.36° Fac Bo: Coronal: 1.33 ± 0.82 mm; Apical: 1.50 ± 0.72 mm; Angular: 3.20 ± 2.70°
Verhamme et al. (2013) [31]	3D Deviations (Mean ± SD): Angular: 2.44° ± 1.20° Coronal 3D: 1.40 ± 0.49 mm Apical 3D: 1.58 ± 0.51 mm Depth: 0.92 ± 0.44 mm Mesio-Distal Plane (Mean ± SD): Angular: 1.76° ± 1.02° Coronal Linear: 0.72 ± 0.61 mm Apical Linear: 0.94 ± 0.66 mm Max Angular: 3.41° Max Coronal Linear: 2.42 mm Max Apical Linear: 2.84 mm
Cassetta et al. (2014) [32]	Total Error: Global Coronal: 1.10 ± 0.39 mm Angular: 4.33 ± 1.42° Random Error: Global Coronal: 0.74 ± 0.30 mm Angular: 3.61 ± 0.88° Systematic (Guide Positioning) Error: Global Coronal: 0.36 ± 0.43 mm (Statistically significant, p = 0.038) Angular: 0.72 ± 1.03° (Not statistically significant)
Arısan et al. (2010) [33]	System I (Materialise): Bone-supported: Angular 5.0° ± 1.66°; Coronal 1.70 ± 0.52 mm; Apical 1.99 ± 0.64 mm. Tooth-supported: Angular 3.5° ± 1.38°; Coronal 1.31 ± 0.59 mm; Apical 1.62 ± 0.54 mm. Mucosa-supported: Angular 4.23° ± 0.72°; Coronal 1.24 ± 0.51 mm; Apical 1.4 ± 0.47 mm. System II (Nobel Biocare): Bone-supported: Angular 4.73° ± 1.28°; Coronal 1.56 ± 0.25 mm; Apical 1.86 ± 0.4 mm. Tooth-supported: Angular 3.39° ± 0.84°; Coronal 0.81 ± 0.33 mm; Apical 1.01 ± 0.40 mm. Mucosa-supported: Angular 2.9° ± 0.39°; Coronal 0.7 ± 0.13 mm; Apical 0.76 ± 0.15 mm.
Kraft et al. (2020) [34]	Angular Deviation: 3.60° ± 2.84° (PGS) vs. 5.36° ± 4.53° (FGS) Linear Global at Cervix: 1.34 ± 0.99 mm (PGS) vs. 1.26 ± 0.57 mm (FGS) Linear Global at Apex: 1.97 ± 1.04 mm (PGS) vs. 2.50 ± 1.67 mm (FGS) Linear Facial-Palatal at Cervix: 0.53 ± 0.36 mm (PGS) vs. 0.48 ± 0.51 mm (FGS) Linear Facial-Palatal at Apex: 1.09 ± 0.76 mm (PGS) vs. 1.86 ± 1.82 mm (FGS) Linear Mesio-Distal at Cervix: 0.37 ± 0.33 mm (PGS) vs. 0.39 ± 0.36 mm (FGS) Linear Mesio-Distal at Apex: 0.77 ± 0.73 mm (PGS) vs. 0.66 ± 0.71 mm (FGS) Linear Apico-Coronal at Cervix: 1.04 ± 1.05 mm (PGS) vs. 0.90 ± 0.63 mm (FGS) Linear Apico-Coronal at Apex: 1.04 ± 1.05 mm (PGS) vs. 1.01 ± 0.64 mm (FGS)
Alqutaibi et al. (2025) [35]	Overall Mean Deviations (FG vs. PG): Entry Deviation: 0.63 mm (FG) vs. 0.66 mm (PG) Apex Deviation: 0.81 mm (FG) vs. 0.84 mm (PG) Angular Deviation: 2.11° (FG) vs. 2.17° (PG) Statistical Summary: The FG approach resulted in statistically significantly higher overall trueness (*p*-values: Entry = 0.030, Apex = 0.013, Angle = 0.036). Differences at individual implant sites were not consistently significant.
D’addazio et al. (2022) [36]	Overall (n = 48): Platform Deviation (A): 0.803 ± 0.433 mm Apex Deviation (B): 1.20 ± 0.484 mm Depth Deviation (C): 1.22 ± 0.65 mm Angular Deviation (D): 4.186 ± 1.486° Group A (DICOM-DICOM) vs. Group B (DICOM-STL): No statistically significant differences for any parameter (*p* > 0.05). Mandible vs. Maxilla: Statistically significant differences, with mandibular implants showing greater accuracy.
Cassetta et al. (2013) [37]	Global Coronal Deviation: 1.59 ± 0.68 mm (Fixed), 1.55 ± 0.59 mm (Not Fixed) Global Apical Deviation: 2.07 ± 0.88 mm (Fixed), 2.05 ± 0.89 mm (Not Fixed) Angular Deviation: 4.11 ± 2.40° (Fixed), 5.46 ± 3.38° (Not Fixed) Depth Deviation: 0.98 ± 0.74 mm (Fixed), 0.63 ± 0.43 mm (Not Fixed) Lateral Deviation: 1.06 ± 0.63 mm (Fixed), 1.36 ± 0.58 mm (Not Fixed) Calculated Mean Intrinsic Error (Angular): 2.57°

**Table 5:** Comparative framework of the two primary reference systems for assessing CGSIS accuracy.

Characteristic	CBCT-Based Assessment	IOS/STL-Based Assessment
Primary Data Source	Post-operative CBCT scan.	Intraoral scan (IOS) with connected scan body (or laboratory scan of a master cast).
Reference System	Superimposition of pre-op CBCT (with plan) and post-op CBCT.	Superimposition of planned STL (with virtual scan body) and post-op STL (with actual scan body).
Main Advantages	Direct visualization of the implant within the bone. Validates position relative to vital anatomic structures (e.g., inferior alveolar nerve, maxillary sinus).	High surface precision of optical scanning, avoiding CBCT scatter artifacts. Radiation-free. Efficient, chairside workflow.
Main Sources of Error	Metal scatter artifacts obscuring implant axis. Voxel size and segmentation accuracy. Error in surface-based registration of pre-/post-op volumes.	Infers apical position from the planned axis; does not detect implant bending/deflection. Dependent on scan body connection precision and scan accuracy.
Type of Accuracy Assessed	Bone-level accuracy. Answers: “Was the implant placed correctly in the bone according to the plan?”	Prosthetic-driven (restorative) accuracy. Answers: “Is the implant platform in the correct position to receive the planned prosthesis?”
Clinical Question Addressed	Safety related to anatomy; suitability for immediate loading in terms of primary stability.	Restorability; passivity of the final prosthesis; aesthetic emergence profile.

## Data Availability

No new data were created or analyzed in this study.

## Supplementary Materials

The following supporting information can be downloaded at: https://www.mdpi.com/article/10.3390/dj14010043/s1, Supplementary File S1: PRISMA-ScR Checklist.

## Abbreviations

The following abbreviations are used in this manuscript:

CGSIS	Computer-guided static implant surgery
PRISMA-ScR	Preferred Reporting Items for Systematic reviews and Meta-Analyses extension for Scoping Reviews
CBCT	Cone-Beam Computed Tomography
IOS	Intraoral Scanner
STL	Standard Tessellation Language/Stereolithography
RCT	Randomized Controlled Trial.
JBI	Joanna Briggs Institute
PCC	Population, Concept, Context
IQR	Interquartile Range
s-CAIS	Static computer-assisted implant surgery
r-CAIS	Robotic-assisted implant surgery
